# Time Length of Negativization and Cycle Threshold Values in 182 Healthcare Workers with Covid-19 in Milan, Italy: An Observational Cohort Study

**DOI:** 10.3390/ijerph17155313

**Published:** 2020-07-23

**Authors:** Lisa Cariani, Beatrice Silvia Orena, Federico Ambrogi, Simone Gambazza, Anna Maraschini, Antonella Dodaro, Massimo Oggioni, Annarosa Orlandi, Alessia Pirrone, Sara Uceda Renteria, Mara Bernazzani, Anna Paola Cantù, Ferruccio Ceriotti, Giovanna Lunghi

**Affiliations:** 1 Fondazione IRCCS Ca’ Granda Ospedale Maggiore Policlinico, Microbiology Unit, 20122 Milan, Italy; anna.maraschini@policlinico.mi.it (A.M.); antonella.dodaro@policlinico.mi.it (A.D.); pirronealessia97@gmail.com (A.P.); 2 Specialization School in Microbiology and Virology, University of Milan, 20142 Milan, Italy; 3 Laboratory of Medical Statistics and Biometry, Giulio A. Maccacaro, Department of Clinical Sciences and Community Health, Campus Cascina Rosa, University of Milan, 20133 Milan, Italy; Federico.Ambrogi@unimi.it (F.A.); simone.gambazza@policlinico.mi.it (S.G.); 4 UOC Direzione Professioni Sanitarie, Fondazione IRCCS Ca’ Granda Ospedale Maggiore Policlinico, 20122 Milan, Italy; 5 Fondazione IRCCS Ca’ Granda Ospedale Maggiore Policlinico, Virology Unit, 20122 Milan, Italy; massimo.oggioni@policlinico.mi.it (M.O.); annarosa.orlandi@policlinico.mi.it (A.O.); sara.ucedarenteria@policlinico.mi.it (S.U.R.); giovanna.lunghi@policlinico.mi.it (G.L.); 6 Fondazione IRCCS Ca’ Granda Ospedale Maggiore Policlinico, Responsabile Infermieristico area Direzione Medica di Presidio, Coordinamento dipartimenti clinici, Servizio Prevenzione Igiene Ospedaliera, 20122 Milan, Italy; mara.bernazzani@policlinico.mi.it; 7 Fondazione IRCCS Ca’ Granda Ospedale Maggiore Policlinico, Direzione Medica di Presidio, 20122 Milan, Italy; annapaola.cantu@policlinico.mi.it; 8 Fondazione IRCCS Ca’ Granda Ospedale Maggiore Policlinico, Romeo ed Enrica Invernizzi Paediatric Research Centre, Department of Biosciences, University of Milan, 20122 Milan, Italy; 9 Fondazione IRCCS Ca’ Granda Ospedale Maggiore, Clinical Laboratory, 20122 Milan, Italy; ferruccio.ceriotti@policlinico.mi.it

**Keywords:** SARS-CoV-2, Coronavirus Disease 2019, Reverse Transcription PCR, Cycle Threshold values, health care workers

## Abstract

*Background*: Coronavirus Disease 2019 (COVID-19) has rapidly spread worldwide, becoming an unprecedented public health emergency. Rapid detection of Severe Acute Respiratory Syndrome Coronavirus 2 (SARS-CoV-2) suspected cases is crucial to control the spread of infection. We aimed to evaluate the time length of negativization from the onset of symptoms in healthcare workers (HCWs) with COVID-19, and to evaluate significant variations in cycle threshold (CT) values and gene positivity (E, RdRP, and N genes) among positive individuals who returned to work. *Methods*: We retrospectively analyzed a consecutive cohort of 182 SARS-CoV-2-positive HCWs in Milan, from 16 March to 30 April 2020. Nasopharyngeal swabs were tested by RT-PCR. *Results*: Asymptomatic HCWs were 17.6% (32/182), and 58 healed at 30 April 2020. The median time length of negativization was 4 weeks (35% of symptomatic versus 40% of asymptomatic HCWs). Four HCWs, healed at 30 April, turned positive within three weeks during controls set up in the work unit. Three-gene positivity had the greatest variability, and increasing CT values from single- to three-gene positivity among all age groups were observed. *Conclusions*: Self-isolation longer than two weeks and prolonged follow-up periods for the staff returning to work after COVID-19 could be the most suitable choices to counter the SARS-CoV-2 spread. Further studies are needed to investigate infectiousness profiles among positive individuals.

## 1. Introduction

The novel Severe Acute Respiratory Syndrome Coronavirus 2 (SARS-CoV-2) is the aetiological agent of the severe acute respiratory syndrome named coronavirus disease 2019 (COVID-19). Because of its rapid and uncontrolled global spread, COVID-19 was declared a pandemic by the World Health Organization with a total of 3,672,238 cases and 254,045 deaths since the first cases were reported in Wuhan (Hubei Province, China) in December 2019 (data updated to 7 May 2020) [1,2].

SARS-CoV-2 is a β-coronavirus, enveloped, non-segmented, and positive-sense single-stranded RNA virus, belonging to the subfamily of the *Orthocoronavirinae* [3]. COVID-19 shows similarities in symptoms with earlier β-coronavirus diseases, such as fever, dry cough, dyspnea, fatigue, headache, sputum production, hemoptysis, lymphopenia, and bilateral ground-glass opacities on chest computed tomography scans. It is known that sometimes patients with SARS-CoV-2 infection also develop gastrointestinal symptoms like diarrhea, unlike patients with Middle East Respiratory Syndrome Coronavirus (MERS-CoV) and SARS-CoV [4,5]. The course of COVID-19 is long, and the disease is highly contagious even during the incubation period (an estimated period ranging from 2 to 14 days, most commonly 5.2 days). Furthermore, asymptomatic carriers of SARS-CoV-2 account for 1–18% of the laboratory-confirmed cases of COVID-19 [6,7], even though studies on estimation of asymptomatic prevalence report higher rates up to 30.8% [8,9,10].

Italy became a hot spot for the COVID-19 pandemic after China, with 223,096 cases and 31,368 deaths recorded by mid-May 2020. Among these, healthcare workers (HCWs) counted for 11% of all COVID-19 cases in Italy [6,11]. During SARS and MERS outbreaks, the spread of infection to HCWs was a significant concern. Systematic implementation of public health measures, such as active case detection, rapid case and contact isolation, as well as strict application of disease prevention and control procedures, have been successful in epidemic control. In addition, rapid collection and testing of appropriate samples of COVID-19 suspected cases (those with typical respiratory symptoms or in the case of documented close contact with SARS-CoV-2 patients, co-workers, or relatives) are crucial for clinical management of healthcare staff, and to avoid the collapse of the healthcare system [12,13]. 

Global guidelines recommend nucleic acid amplification tests, such as real-time reverse transcription polymerase chain reaction (RT-PCR), as the standard of reference for the diagnosis of SARS-CoV-2 infection. The viral genes detected include: nucleocapsid (N) and envelope (E) proteins [14,15,16] that play a key role in viral self-assembly, RNA-dependent RNA Polymerase (RdRP) [15], and spike (S) glycoprotein that interacts with the host cell’s ACE2 receptors [17,18].

In this study, we retrospectively analyzed RT-PCR test results of a consecutive cohort of HCWs from the Fondazione IRCCS Ca’ Granda Ospedale Maggiore Policlinico in Milan, Italy. Our aims were to evaluate the time length of negativization from the onset of symptoms, and to evaluate variations of cycle threshold (CT) values and gene positivity (E, RdRP, and N genes) among positive individuals who returned to work.

## 2. Materials and Methods

### 2.1. Study Population

**Figure 1:**
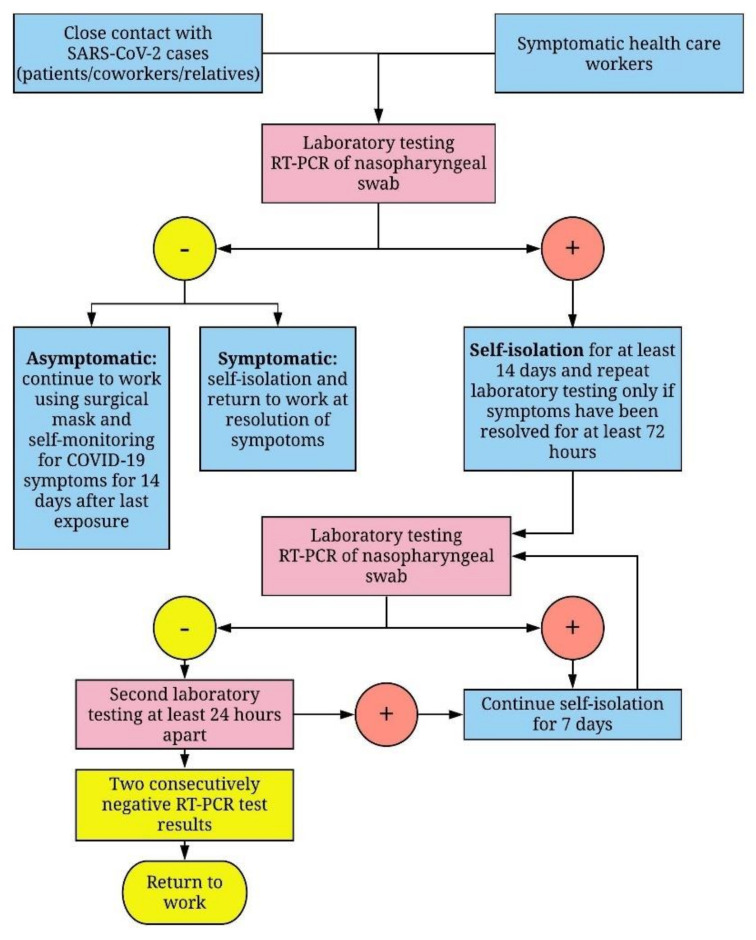
Flowchart for the management of healthcare workers (HCWs) with suspected or confirmed Severe Acute Respiratory Syndrome Coronavirus 2 (SARS-CoV-2) infection. According to the procedures implemented by Fondazione IRCCS Ca’ Granda Ospedale Maggiore Policlinico in Milan, all HCWs suspected of SARS-CoV-2 infection must be tested by RT-PCR analysis of nasopharyngeal swabs and, if necessary, put into isolation for at least 14 days. The following criteria had to be met for discontinuation of quarantine: (a) resolution of respiratory symptoms and an apyretic state lasting longer than 72 h, and (b) two consecutively negative RT-PCR test results obtained on swabs collected at least 24 h apart. The same procedures were used for asymptomatic HCWs, with the exception of criteria (a).

From 16 March to 30 April 20,201,683 HCWs were tested for SARS-CoV-2 infection according to the procedures available at Fondazione IRCCS Ca’ Granda Ospedale Maggiore Policlinico in Milan, Italy (Figure 1). All HCWs had common respiratory infection symptoms, or had close contact with SARS-CoV-2-positive cases (patients, co-workers, or relatives).

### 2.2. RT-PCR Detection

Nasopharyngeal samples were collected with flocked swabs in 3 mL of universal transport medium. After collection, total RNA was extracted using a Seegene STARMag 96 × 4 Universal Cartridge Kit, an automatic nucleic acid purification system. The presence of SARS-CoV-2 was analyzed by RT-PCR. SARS-CoV-2 envelope (E) proteins, nucleocapsid (N) proteins, and RNA-dependent RNA Polymerase (RdRP) gene fragments were detected by a Seegene Allplex^TM^ 2019 n-CoV assay. The first assessment of kit performance by the manufacturer demonstrated a specificity of 100% and a sensitivity of 100 RNA copies/PCR reactions [19]. The conditions for amplification were 50 °C for 15 min, 95 °C for 15 min, followed by 45 cycles at 94 °C for 15 sec, and 58 °C for 30 sec. A cycle threshold value of less than 40 is defined as a positive test, while a CT value of 40 or more is defined as a negative test. A CT value less than 40 for only one of the three targets is defined as weak positive, whereas a CT value less than 40 for two or more targets is considered positive. The CT value is inversely proportional to the amount of viral nucleic acid in specimens (meaning that a lower CT value indicates a higher amount of virus) and it can be used to estimate viral load.

### 2.3. Statistical Analysis

Metrics were reported as mean and standard deviation, or counts and percentage. Considering HCWs recovered by 30 April 2020 in the sample under study, the proportion of tests turned negative was represented by means of cumulative incidence plots. Only healthcare professionals who turned negative were analyzed. Time was discretized in weeks: 1 means from beginning of symptoms (or the first positive test in asymptomatic subjects) and the next 7 days, 2 means from day 8 since the beginning of symptoms (or the first positive test in asymptomatic subjects) through to the 14th day, and so on. This was due to the fact that the exact days of test administration to evaluate the negativity were affected, not only by clinical considerations, but also for logistic reasons. Moreover, after a negative test, it is not known the exact time when negativization occurred.

The association between categorical variables for HCWs who tested SARS-CoV-2 positive was assessed using chi-square statistics. A non-parametric ANOVA was used to analyze the difference in the viral load at baseline between subjects.

For healthcare workers healed during the period of study, the association of the gene numbers (positivity for three, A, versus two, B, genes) with the viral load after controlling for basal CT values was assessed by the analysis of covariance.

For all analyses, *p*-values were two-sided, and *p* < 0.05 was considered to be statistically significant. All of the current analyses were performed using R Core Team, version 3.6.2. (R Foundation for Statistical Computing, Vienna, Austria).

## 3. Results

### 3.1. General Description

A total of 2443 nasopharyngeal swabs were collected from 1683 HCWs. Among these individuals, 1081 were women (64.2%). A total of 182 out of 1683 (10.8%) were SARS-CoV-2 positive, and 105 (57.7%) of these were female. Considering all HCWs tested, 12.8% of males tested positive while the percentage of females was 9.4%. The mean age of SARS-CoV-2-positive HCWs was 43.5 (range 21.4–70.3, I-III quartile 31.5–54.1) years. The majority of HCWs testing positive (150, 82.4%) presented with symptoms.

Twenty-seven HCWs out of 182 (14.8%) were tested for SARS-CoV-2 infection because of close contact with COVID-19 cases, and resulted negative at the first test. They repeated the test at the beginning of respiratory symptom onset (with a mean of 5.6 days after the negative RT-PCR results) and the nasopharyngeal specimens resulted positive for SARS-CoV-2.

At the time of writing, we have complete information about negativization on 58 out of 182 HCWs healed by April 30, 2020 (absence of clinical symptoms and two negative RT-PCR test results during the period of study). Among these, 48 HCWs were symptomatic at the moment of nasopharyngeal swabs, 10 HCWs were asymptomatic, and only 1 developed symptoms three days after specimen collection. Table 1 reports the demographic characteristics of HCWs who tested positive, as well as HCWs who healed by April 30, 2020. The healthcare workers most affected were physicians (43%). Overall, the main symptoms reported by 182 HCWs were: fever (48.3%), cough (20.7%), headache (19%), rhinitis (17.2%), sore throat/tracheitis (15.5%), muscle/joint pain (12.1%), anosmia (12.1%), ageusia (10.4%), conjunctivitis (3.5%), dyspnea (3.5%), and gastrointestinal symptoms (1.7%).

### 3.2. Time at Which Subjects Became Test-Negative

Considering the 58 subjects healed by 30 April 2020, the time length of negativization was calculated from the beginning of symptom onset to the two consecutively negative RT-PCR test results, collected at least 24 h apart after the disappearance of symptoms. In the case of asymptomatic HCWs, the calculation was carried out from the first positive RT-PCR test result.

The majority of subjects became negative at three weeks or later. More than 70% of HCWs with symptoms became test-negative within four weeks; this proportion rises to 80% in the asymptomatic group. Moreover, 29.2% of symptomatic versus 20% of asymptomatic HCWs became test-negative at five weeks.

In addition, it is worth reporting that four out of 58 (6.9%) HCWs, who went back to work after the two consecutive negative tests, were found positive in a RT-PCR test for SARS-CoV-2 during controls set up in the unit of work within 3 weeks.

### 3.3. Analysis of CT Values of RT-PCR Tests

**Figure 2:**
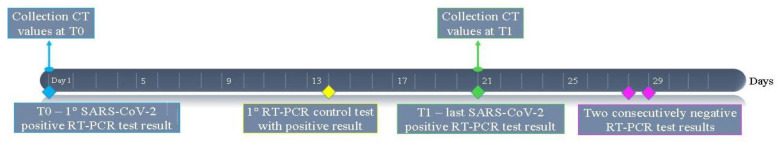
Timeline of cycle threshold (CT) value collection. An example according to a time length of negativization of 4 weeks.

We collected cycle threshold values of the first SARS-CoV-2-positive nasopharyngeal swabs (T0) for all 182 HCWs and CT values at one week before the two negative RT-PCR tests (T1) for the 58 subjects who healed by 30 April 2020 (Figure 2).

No evidence of association was found in the distribution of gene positivity among males and females (*p* = 0.8973) in the 182 HCWs who tested positive.

**Figure 3:**
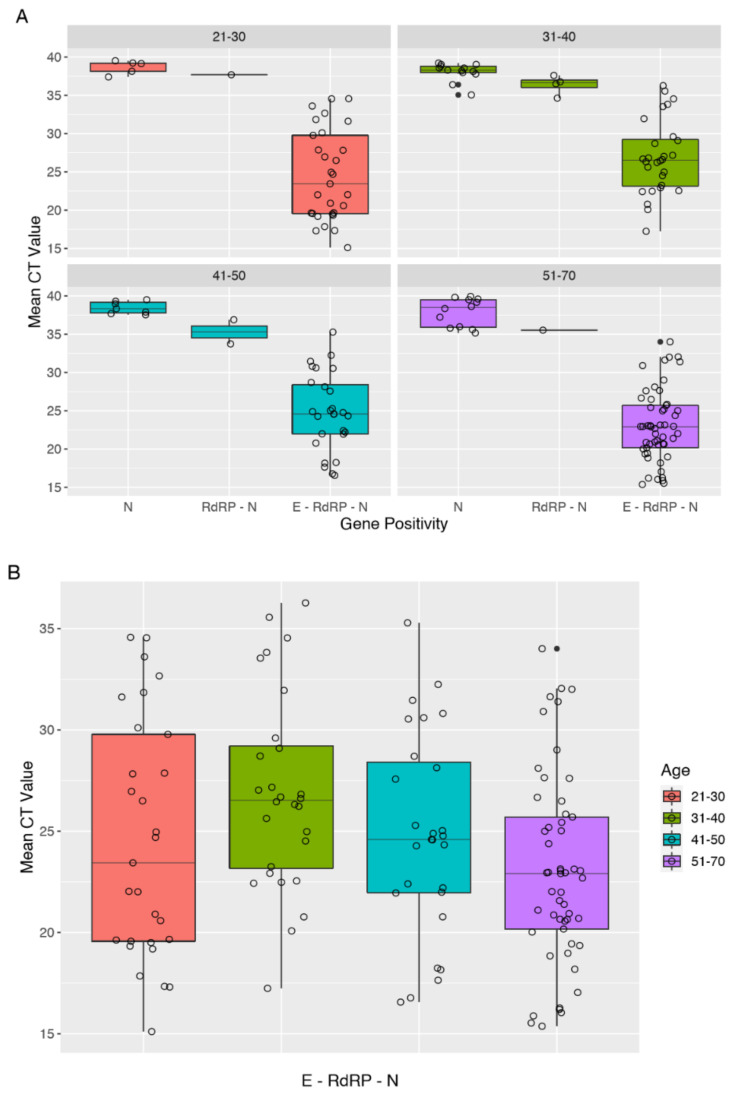
Distribution of gene positivity and mean cycle threshold (CT) values among different age groups. (**A**) Box-plot of mean viral load (cycle threshold) across different ages according to gene positivity. Empty circles represent a single observation for each gene. (**B**) Box-plot of mean viral load at baseline for the gene positivity E–RdRP–N among different age-groups. Empty circles represent a single observation for each age group.

Looking at the distribution of gene positivity and mean CT values among four age-groups, we observed a constant pattern of viral load across different age-groups (Figure 3A). Particularly, E–RdRP–N shows the greatest variability (Figure 3B); the mean CT significantly differs across age groups (*p* = 0.01637). In addition, we observed increasing CT values from single-gene to three-gene positivity among all age groups. N–RdRP shows CT values very close to N, even though they are reported as positive and single-gene positive tests, respectively.

**Figure 4:**
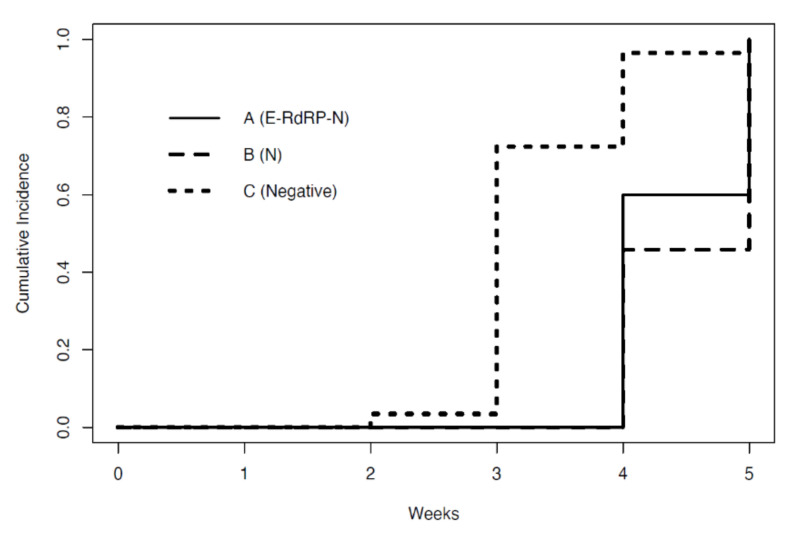
Time at which HCWs, stratified by gene positivity, became negative.

Furthermore, the 58 HCWs healed by April 30, 2020 were divided into three groups based on the gene positivity at T1. Group A includes HCWs with positivity to all three genes (E–RdRP–N). Groups B and C include HCWs with single-gene positivity and those who tested negative at T1, respectively (Table 2). There was no evidence of variation of the viral load at T0 (*p* = 0.1307). However, after adjusting for the baseline viral load, there was a statistically significant difference in viral load at T1 between groups A and B (F(2,26) = 16.51, *p* < 0.001)). Figure 4 shows that the time length of negativization of groups A and B varied from 4 to 5 weeks. In contrast, the majority of HCWs in group C became negative earlier.

## 4. Discussion

Health-care-associated amplification of transmission is alarming, as is always the case for emerging infections. Since the first positive laboratory test result in Italy, on 21 February 2020, the Italian healthcare system has been fighting COVID-19 [20].

In the present study, we analyzed 2443 nasopharyngeal swabs from 1683 HCWs by molecular laboratory testing for suspected SARS-CoV-2 infection in a large university hospital in Milan, showing 10.8% positive HCWs. Overall, the majority of HCWs with COVID-19 were physicians, and the main reported symptoms were fever, cough, and headache. In contrast to Rothan’s study, only one subject experienced gastrointestinal symptoms [5].

The prevalence of asymptomatic carriers (17.6%) among HCWs with COVID-19 is similar to national data reported by the Istituto Superiore di Sanità, which detected 17.1% of asymptomatic carriers in 53,919 SARS-CoV-2-positive cases [6].

We observed that 35% of subjects with symptoms, versus 40% without symptoms, had recovered at four weeks. Absence of symptoms corresponds to an undefined onset of COVID-19, and the time length of negativization of asymptomatic individuals could be longer. Our findings are supported by a recent study conducted by Zhou and colleagues [21], in which they detected SARS-CoV-2 for a median of 20 days, up to 37 days after symptom onset, in respiratory samples. Taken together, these results show how the original recommendation of 14 days of self-isolation was probably an underestimate of the time needed to recover.

We also reported that 14.8% (27/182) of HCWs tested positive to SARS-CoV-2 only at the beginning of symptom onset, within 5.6 days after the first negative RT-PCR test result. Our data seem to differ from a recent study in which Xi and colleagues suggested that viral shedding could begin two or three days before symptom onset [22]. The proportions of asymptomatic and initially negative cases represent major issues for the control of outbreaks, both in hospital and in social environments, as they are able to infect a large amount of the population due to the highly contagious nature of SARS-CoV-2. RT-PCR assay plays a central role in the detection of COVID-19 infection, but it might be insufficient to accurately estimate the overall infected population.

As regards the mean CT values at T0 for 182 positive HCWs, there was no significant association in viral loads between sex, nor a statistically significant variation among age groups. HCWs with E–RdRP–N gene positivity showed the highest viral loads in every age group. Furthermore, we observed a close similarity between viral loads of positive subjects with two-gene positivity (N+RdRP), and subjects with single-gene positivity (N). It could be of interest to further investigate differences and similarities between infectiousness profiles.

Additionally, we observed that about 70% of HCWs having the lowest viral loads became test-negative at three weeks, while about 50% of HCWs from groups A and B became test-negative after at least one more week.

The case of the four HCWs who tested positive within of 3 weeks of RT-PCR tests for SARS-CoV-2 and after two consecutive negative results suggested that in some individuals viral shedding may continue at a very low titer for some time or that RT-PCR had detected only RNA fragments without clinical significance. A growing number of studies have reported a significant portion of patients turning positive, suggesting that a longer observation period should be considered for COVID-19 patients [23].

This study has several limitations. Firstly, it analyzed a limited cohort. Larger studies on HCWs should be done to improve the management of healthcare staff with COVID-19, and consequently in the entire population, in order to better control the viral outbreak. Additionally, we were able to consider healed, just over 30% of positive HCWs with respect to the period of study. Secondly, symptom details rely on patient self-assessment at the moment of specimen collection for SARS-CoV-2 diagnosis. Wrong epidemiological data can lead to an improper estimation of COVID-19 incubation periods and asymptomatic prevalence. Thirdly, the RT-PCR test can be affected by many external factors, such as specimen source, sampling collection (sample quality) and timing (a virus may not be detected at early stages of infection), and kit performance (specificity and sensitivity).

RT-PCR is one of the most well-established laboratory diagnosis tests for use in a new viral pandemic, even though several authors have remarked on the poor sensitivity of this method [13,24]. The combination of several tools, like a detailed anamnestic report, and molecular and serological SARS-CoV-2 diagnosis, could be considered to be the next best laboratory approach in order to reduce the rate of false-negative RT-PCR results. Furthermore, it is known that a significant proportion of individuals infected by SARS-CoV-2 presented with gastrointestinal disorders [23,25]. Consequently, it seems to be important to integrate SARS-CoV-2 detection with stool samples, especially considering that oral–fecal transmission could occur even after viral clearance in the airways [5,26]. All these considerations suggest the need for an improved diagnostic process, to identify and isolate at early stages all SARS-CoV-2-positive HCWs, both symptomatic and asymptomatic.

Furthermore, viral particles detected by RT-PCR are not necessarily correlated with the ability of transmission. It could be important to estimate the infectiousness of a patient with a CT value close to a value considered negative, and to perform a cell culture. Indeed, recent studies reported that a live virus could no longer be cultured from 8 days after symptom onset, and that the infectiousness profile of SARS-CoV-2 seems to be more like influenza than SARS [21,27].

## 5. Conclusions

Self-isolation longer than two-weeks and a prolonged follow-up period for staff who have returned to work after testing positive for COVID-19 could be the most suitable choices to counter the spread of SARS-CoV-2. According to our results, we suggest an isolation period of 28 days. Further studies are needed to investigate the correlation between CT values and SARS-CoV-2 infectivity.

## Figures and Tables

**Table 1:** Demographic characteristics of positive HCWs (*n* = 182) and healed HCWs (*n* = 58) who returned to work.

	Positive HCW	Healed HCW at 30 April 2020
*n*	182	58
Age, year	43.5 (12.9)	42.2 (13.8)
Sex		
Male	77 (42.3%)	31 (53.4%)
Female	105 (57.7%)	27 (46.6%)
Professional role		
Physicians	71 (39%)	25 (43%)
Nurses	48 (26.5%)	13 (22%)
Non-medical workers	33 (18%)	9 (16%)
Health technicians	22 (12%)	8 (14%)
Sanitary Operator Partners	8 (4.5%)	3 (5%)

Data are reported as mean (SD) or count (percentage).

**Table 2:** Viral loads among three different groups of HCWs. Data are reported as mean (SD).

Group	*n*	Mean CT T0	Mean CT T1
A: Positive (E–RdRP–N)	5	22.60 (8.29)	33.60 (2.18)
B: Single-gene positive (N)	24	22.06 (4.40)	38.03 (1.43)
C: Negative	29	25.08 (6.02)	-
